# Diversity, equity, and inclusion in arrhythmia care: a European Heart Rhythm Association survey

**DOI:** 10.1093/europace/euag144

**Published:** 2026-06-11

**Authors:** Mark T Mills, Maura M Zylla, Gabor Z Duray, Jarkko Karvonen, Melanie A Gunawardene, Christian-Hendrik Heeger, Katarzyna Malaczynska-Rajpold, Michal Mazurek, Martina Nesti, Diego Penela, Laura Perrotta, Martin H Ruwald, Daniel Scherr, Matteo Anselmino, Julian K Chun

**Affiliations:** Department of Cardiology, Northern General Hospital, Sheffield Teaching Hospitals NHS Foundation Trust, Sheffield, UK; Department of Cardiology, Heidelberg Center of Heart Rhythm Disorders, Medical University Hospital, Im Neuenheimer Feld 410, Heidelberg, Germany; Department of Cardiology, Central Hospital of Northern Pest-Military Hospital, Budapest, Hungary; Heart and Vascular Center, Semmelweis University, Budapest, Hungary; Heart and Lung Center, Helsinki University Hospital and University of Helsinki, Helsinki, Finland; Cardioangiologisches Centrum Bethanien, Agaplesion Markus Krankenhaus, Frankfurt am Main, Germany; Department of Rhythmology, Cardiology and Internal Medicine, Asklepios Klinik Hamburg Altona, Hamburg, Germany; Heart, Lung and Critical Care, Royal Brompton Hospital, Guy's and St Thomas’ NHS Foundation Trust, London, UK; Cardiology Department, Lister Hospital, East and North Hertfordshire NHS Trust, Stevenage, UK; 1st Department of Cardiology and Angiology, Silesian Center for Heart Diseases, Zabrze, Poland; Cardiology Department, Fondazione Toscana Gabriele Monasterio, Pisa, Italy; Department of Cardiovascular Medicine, IRCCS Humanitas Research Hospital, 20089 Rozzano, Milan, Italy; Arrhythmia Unit, Department of Cardiology, Careggi University Hospital, Florence, Italy; Division of Electrophysiology, Heart Center Copenhagen, Rigshospitalet, Copenhagen, Denmark; Division of Cardiology, Medical University Graz, Graz, Austria; Division of Cardiology, Cardiovascular and Thoracic Department, ‘Città della Salute e della Scienza di Torino’ Hospital, Corso Dogliotti 14, 10126 Turin, Italy; Department of Medical Sciences, University of Turin, Turin, Italy; Cardioangiologisches Centrum Bethanien, Agaplesion Markus Krankenhaus, Frankfurt am Main, Germany

**Keywords:** Diversity, Equity, Inclusion, Arrhythmia, Electrophysiology, Pacing, Ablation, Sex, Gender, Ethnicity

## Abstract

**Aims:**

Disparities in arrhythmia care are increasingly recognized, yet remain incompletely characterized across the patient pathway. This European Heart Rhythm Association (EHRA) survey explored clinician-reported perceptions of inequity across diagnosis, pharmacological management, procedural referral, and follow-up.

**Methods and results:**

A 30-question survey was disseminated via the EHRA between November 2025 and January 2026, with 212 responses from professionals across 35 countries. Respondents were predominantly consultant electrophysiologists (67.5%), with 39.6% identifying as female. Most (68.4%) reported no prior training in equity or inclusive care. Across the arrhythmia care pathway, disparities were most frequently attributed to patient vulnerability, particularly cognitive impairment (72.5%), age >80 years (63.8%), and mental health disease (61.3%). Differences related to socioeconomic status, language, and other social factors were also commonly reported. Female sex and minority ethnic background were each reported to influence care in 24.1% of responses. Age >80 years was consistently identified as the strongest determinant of disparities in referral, diagnosis, and outcomes, influencing referral for arrhythmia evaluation (65.8%), catheter ablation (77.9%), and outcomes following ablation (68.1%). Socioeconomic and ethnic factors showed more modest but consistent effects, while sex-based differences were less frequently reported. However, female respondents were more likely than male respondents to report delayed referral (female respondents: 41.8%; male respondents: 19.8%) and late or incorrect diagnosis (49.4 vs. 15.8%) in female patients, as well as delayed referral for catheter ablation (36.5 vs. 10.3%) and device implantation (23.0 vs. 7.0%). Only one-third of respondents (33.3%) felt that current international guidelines adequately address diversity, equity, and inclusion in arrhythmia care.

**Conclusion:**

Clinicians perceive disparities in arrhythmia care across multiple patient and social factors. Whilst age and vulnerability were most frequently perceived to influence care, sex and ethnicity were also recognized by a substantial proportion of respondents. Perceptions varied according to respondent characteristics, with female clinicians more likely to report disparities amongst female patients. Limited training and institutional frameworks highlight opportunities for targeted interventions.

## Graphical Abstract

**Graphical Abstract:**
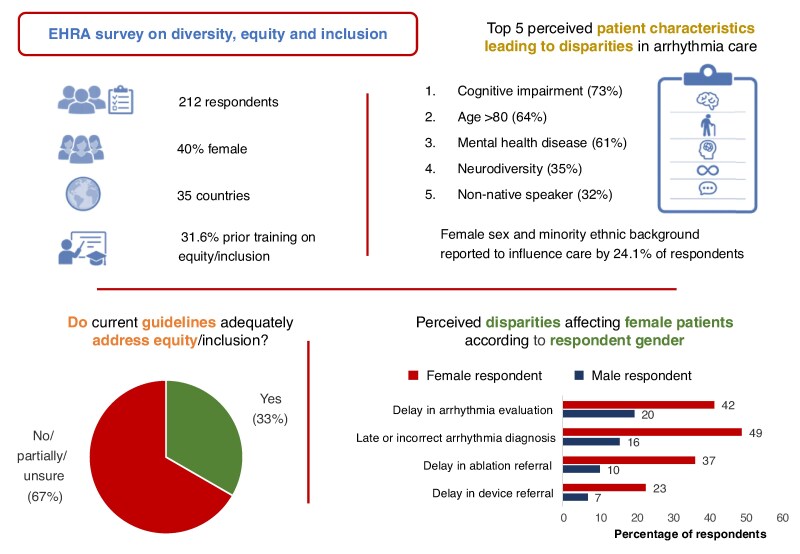

## Introduction

The global prevalence of cardiac arrhythmias is rising, making equitable access to high-quality, patient-centred arrhythmia care increasingly important.^1^ However, substantial disparities in the delivery of guideline-directed therapies and interventional management have been documented, often influenced by patient sex, gender, ethnicity, and socioeconomic status.^1–6^ For example, tachyarrhythmias may be underdiagnosed or misdiagnosed in women, and in atrial fibrillation (AF), women are less likely to receive guideline-directed oral anticoagulation^7^ and are referred for catheter ablation less frequently or at later disease stages, potentially resulting in greater atrial remodelling and reduced long-term procedural success.^8,9^ Similarly, socioeconomic status and ethnic background have been shown to affect access to both catheter ablation and cardiac implantable electronic device therapy.^10–12^ Representation in arrhythmia clinical trials is also skewed, with women and ethnic minorities often under-represented, limiting the generalizability of findings.^13,14^ Certain factors, such as gender identity, language barriers, or other social determinants of health, remain under-investigated regarding their impact on arrhythmia care and clinical outcomes.

To better understand these disparities from the clinician perspective, the European Heart Rhythm Association (EHRA) Scientific Initiatives Committee developed an international survey addressing potential differences in arrhythmia care across diverse patient groups. The survey explored the full patient journey, from initial assessment and diagnosis through pharmacological and interventional treatment to long-term follow-up, and assessed institutional strategies to mitigate barriers. Additionally, it sought to clarify the potential role of professional societies in supporting inclusive, equitable arrhythmia care.

## Methods

### Study design and distribution

A bespoke 30-item questionnaire was developed by the EHRA Scientific Initiatives Committee, comprising electrophysiologists with expertise in clinical arrhythmia management and survey methodology.^15–17^ The questionnaire was reviewed, edited, and approved by all co-authors and was designed to capture clinician-reported perceptions of disparities related to patient sex, gender, ethnicity, age, socioeconomic status, and other social determinants of health. As this was an exploratory survey designed to assess clinician perceptions, formal psychometric validation was not performed. However, the questionnaire underwent iterative review and refinement by members of the EHRA Scientific Initiatives Committee to ensure clarity, relevance, and face validity prior to dissemination. The questionnaire was developed and distributed in English, the working language of EHRA communications and previous EHRA surveys.

For the purpose of this survey, *diversity* refers to variation in patient characteristics and backgrounds, including biological, social, cultural, and demographic factors. *Equity* refers to the principle that individuals should have fair access to healthcare opportunities and resources according to their needs, whereas *inclusion* refers to healthcare environments and processes that enable all individuals to participate fully in their care. The terms *disparity* and *inequity* are used to describe differences in healthcare access, treatment, or outcomes that may be associated with patient characteristics or social factors, although this survey specifically assessed clinician perceptions of such differences rather than objectively measured inequalities. *Sex* refers to biological characteristics, whereas *gender* refers to socially constructed identities, roles, and expressions. *Social determinants of health* encompass non-medical factors influencing health and healthcare access, including socioeconomic status, education, language, geographic location, and social support. Within this article, the term *vulnerability* is used to describe patient groups perceived to be at increased risk of barriers to equitable arrhythmia care.

The final survey was organized into seven thematic sections: (i) respondent characteristics, (ii) perceptions of equity in arrhythmia care; (iii) diagnosis and screening, (iv) pharmacological management, (v) procedural referral and selection, (vi) follow-up and monitoring, and (vii) institutional policy and outlook. Most items used 5-point Likert scales (never, rarely, sometimes, often, always), consistent with prior EHRA surveys, with selected multiple-choice and free-text responses. No adaptive branching logic was employed, and respondents were presented with the same core questionnaire irrespective of their responses. The full survey is provided in Supplementary material online, *Appendix 1*.

In the initial sections, respondents were asked to consider a broad range of patient characteristics, including sex, gender identity, minority ethnic background, language status, cultural or religious beliefs, advanced age (>80 years), cognitive impairment, mental health disease, socioeconomic and educational status, immigration or residency status, neurodiversity, digital literacy, type of health insurance, and geographic location. In subsequent sections, the survey focused on four key characteristics most consistently associated with disparities in the published literature: sex, minority ethnic background, age >80 years, and low socioeconomic status.

The link to the online questionnaire was distributed to the EHRA and EHRA Young EP communities via email, and also promoted via social media, between 28 November 2025 and 19 January 2026. The survey was administered using SurveyMonkey (Momentive Inc., San Mateo, CA, USA), a secure web-based survey platform commonly used for international healthcare research. The survey was open to all healthcare professionals involved in arrhythmia care. Response was voluntary, anonymous, and General Data Protection Regulation (GDPR) compliant. Respondents were permitted to complete the survey only once.

### Statistical analysis

Survey responses were analysed descriptively. Categorical variables are reported as absolute counts and/or percentages, using the number of respondents to each individual question as the denominator. As not all respondents completed every question, denominators varied across analyses and are provided within the corresponding figures. For Likert-scale questions, responses were analysed both as individual categories and, where appropriate, collapsed into grouped categories (e.g. sometimes/often/always) to summarize the proportion of respondents reporting a perceived impact of a given factor. No formal hypothesis testing or comparative statistical analyses were performed, as the survey was exploratory and descriptive in nature. All analyses were performed in SPSS (version 29; IBM).

### Ethical considerations

The survey was conducted in accordance with the principles of the Declaration of Helsinki. Informed consent was requested from respondents at the start of the survey. As this was an anonymous, voluntary survey of healthcare professionals, with no patient-level data collected, formal ethical approval was not required.

## Results

A total of 282 responses were recorded, of which 279 respondents provided consent for analysis. A further 67 responses were excluded due to the lack of study-relevant information (containing no responses beyond respondent baseline demographic data), leaving 212 responses for analysis.

### Respondent characteristics

**Figure 1:**
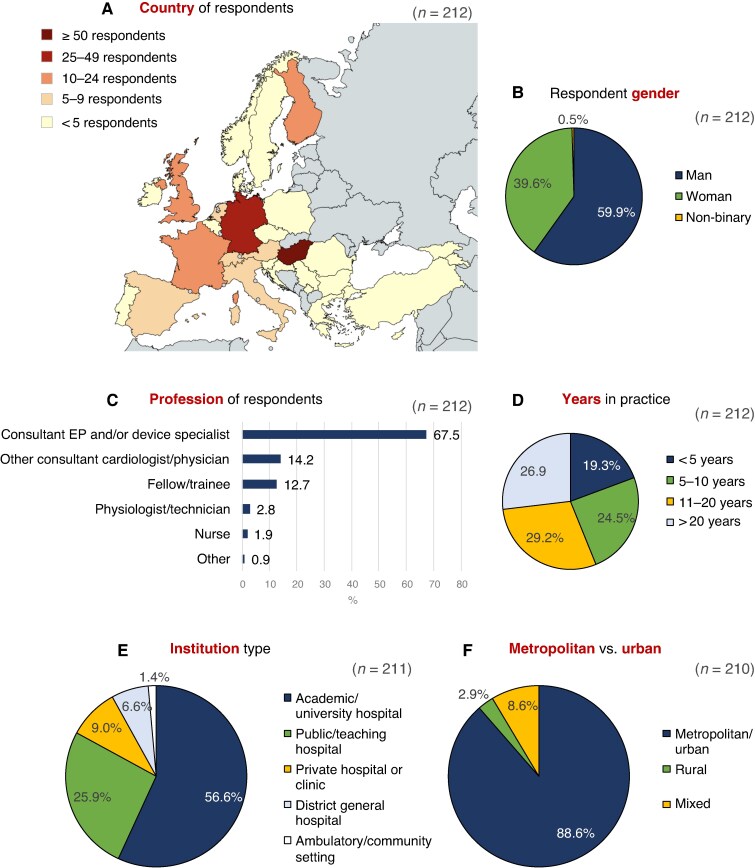
Respondent characteristics. (*A*) Country of respondents (created with MapChart.net). (*B*) Respondent gender. (*C*) Profession of respondents. (*D*) Years in practice. (*E*) Respondent institution. (*F*) Respondent centre location (metropolitan vs. rural).

Respondent characteristics are shown in *Figure 1*. Respondents represented a broad geographic distribution, with the largest contributions from Hungary (24.1%), Germany (14.2%), the UK (8.0%), France (7.5%), and Finland (5.7%) (*Figure 1A*). Gender distribution was 59.9% male, 39.6% female, and 0.5% non-binary (*Figure 1B*). Most respondents were consultant electrophysiologists and/or device specialists (67.5%), followed by consultant cardiologists or other physicians involved in arrhythmia care (14.2%), and fellows or trainees (12.7%), with smaller proportions of physiologists/technicians (2.8%), nurses (1.9%), and other professionals (0.9%) (*Figure 1C*). Experience in arrhythmia practice was broadly distributed, with 19.3% reporting <5 years, 24.5% reporting 5–10 years, 29.2% reporting 11–20 years, and 26.9% reporting >20 years of experience (*Figure 1D*).

Most respondents worked in academic or university hospitals (56.6%), followed by public or teaching hospitals (25.9%), private hospitals or clinics (9.0%), district general hospitals (6.6%), and ambulatory or community settings (1.4%) (*Figure 1E*). The majority practised in metropolitan or urban environments (88.6%), compared with 2.9% in rural areas and 8.6% in mixed settings (*Figure 1F*).

### Training in equity and perceived drivers of inequity

**Figure 2:**
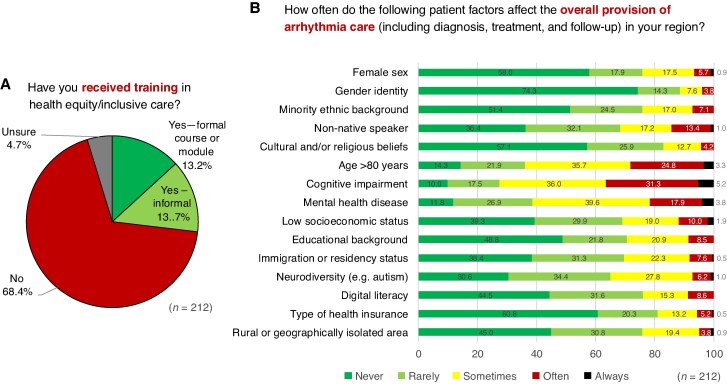
Training in equity and inclusivity, and perceived impact of patient characteristics on arrhythmia care. (*A*) Training in equitable/inclusive care. (*B*) Impact of patient characteristics on overall provision of arrhythmia care.

Most respondents reported no prior training in health equity or inclusive care delivery (68.4%), while 13.2% reported formal training and 13.7% informal training; 4.7% were unsure (*Figure 2A*).

Across the arrhythmia care pathway, cognitive impairment was most frequently reported to influence care, affecting care sometimes, often, or always in 72.5% of responses, followed by age >80 years (63.8%) and mental health disease (61.3%) (*Figure 2B*). Low socioeconomic status (30.8%), non-native speaker (31.6%), immigration or residency status (30.3%), and educational background (29.4%) were also reported to influence care in a substantial proportion of responses (*Figure 2B*).

Female sex and minority ethnic background were each reported to influence care sometimes, often, or always in 24.1% of responses, indicating that these factors were recognized by a meaningful proportion of clinicians. In contrast, gender identity was less frequently reported to influence care (11.4%) (*Figure 2B*).

### Diagnosis and screening

**Figure 3:**
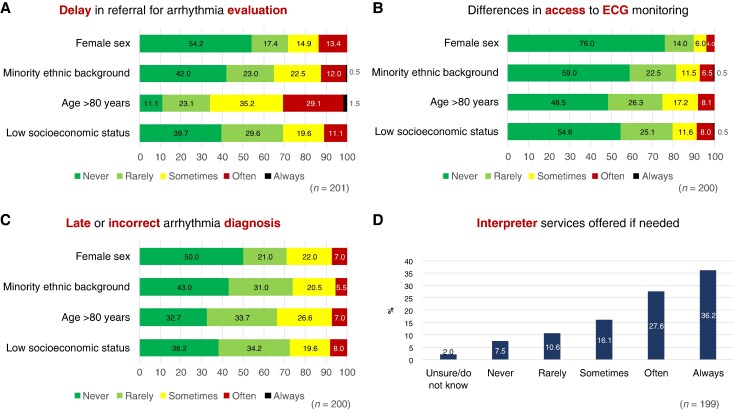
Diagnosis and screening. (*A*) Delay in referral for arrhythmia evaluation. (*B*) Differences in access to ECG monitoring. (*C*) Late or incorrect arrhythmia diagnosis. (*D*) Interpreter services. ECG, electrocardiogram.

Age >80 years was most frequently reported to influence referral for arrhythmia evaluation, with 65.8% of respondents reporting an effect sometimes, often, or always. Lower proportions reported differences for minority ethnic background (35.0%), low socioeconomic status (30.7%), and female sex (28.4%) (*Figure 3A*).

Differences in access to ambulatory electrocardiogram monitoring were less frequently reported overall but remained most evident for age >80 years (25.3%), followed by low socioeconomic status (20.1%), minority ethnic background (18.5%), and female sex (10.0%) (*Figure 3B*).

A similar pattern was observed for late or incorrect diagnosis, with age >80 years influencing diagnosis in 33.7% of responses, followed by female sex (29.0%), low socioeconomic status (27.6%), and minority ethnic background (26.0%) (*Figure 3C*).

Interpreter services for non-native speakers were reported to be available often or always in 63.8% of centres, although a minority of respondents reported limited or no availability (*Figure 3D*).

### Pharmacological management

Tailoring of anti-arrhythmic drug therapy according to patient sex was uncommon, with 7.2% reporting routine tailoring and 46.4% selective use, while 43.3% reported no sex-based adjustment.

**Figure 4:**
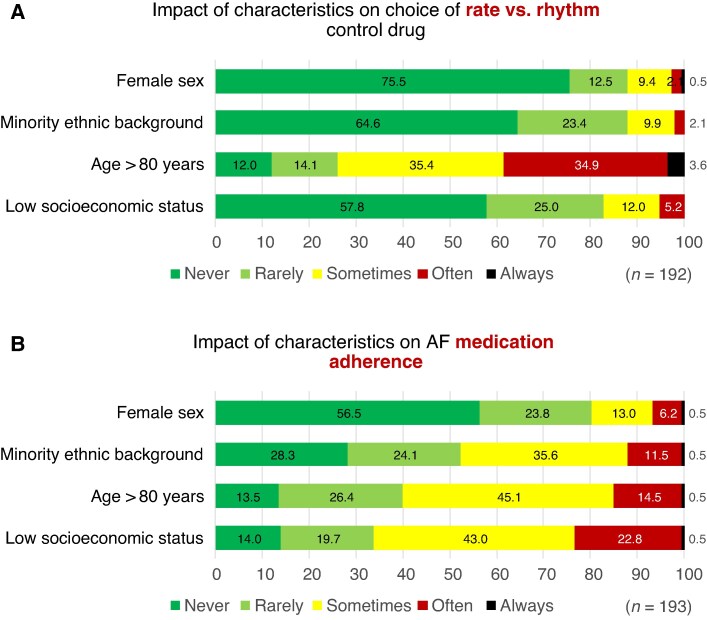
Pharmacological management. (*A*) Impact of patient characteristics on choice of rate vs. rhythm control drug. (*B*) Impact of patient characteristics on AF medication adherence. AF, atrial fibrillation.

Age >80 years was most frequently reported to influence decisions between rate- and rhythm-control strategies, affecting decision-making sometimes, often, or always in 74.0% of responses (*Figure 4A*). In contrast, low socioeconomic status (17.2%), female sex (12.0%), and minority ethnic background (12.0%) were less frequently reported to influence treatment decisions (*Figure 4A*).

Medication adherence was most frequently perceived to be influenced by low socioeconomic status (66.3%) and age >80 years (60.1%), followed by minority ethnic background (47.6%), while female sex was less commonly reported (19.7%) (*Figure 4B*).

### Procedural referral, selection, and complications

**Figure 5:**
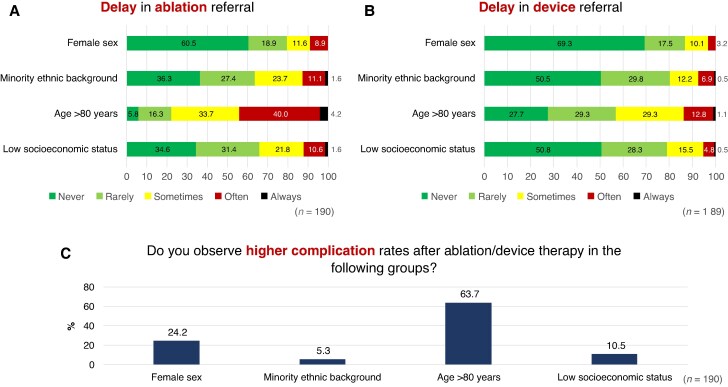
Procedural referral, selection and complications. (*A*) Delay in catheter ablation referral according to patient characteristics. (*B*) Delay in device implantation referral according to patient characteristics. (*C*) Perceived complication rates according to patient characteristics.

Age >80 years was consistently reported as the strongest determinant of reduced or delayed referral for catheter ablation, influencing referral sometimes, often, or always in 77.9% of responses (*Figure 5A*). Differences were also reported for minority ethnic background (36.3%), low socioeconomic status (34.0%), and female sex (20.5%) (*Figure 5A*).

A similar but less pronounced pattern was observed for device implantation, with age >80 years influencing referral in 43.2% of responses, compared with 20.9% for low socioeconomic status, 19.7% for minority ethnic background, and 13.2% for female sex (*Figure 5B*).

Cultural or religious beliefs were reported to influence procedural decision-making occasionally or frequently in 20.6% of responses. Most respondents (75.4%) reported that procedural counselling or technique was never or rarely modified according to patient sex. Higher complication rates were most frequently reported in patients aged >80 years (63.7%), followed by female patients (24.2%), whilst over one-quarter of respondents reported no observed differences in any group (*Figure 5C*).

### Follow-up and outcomes

**Figure 6:**
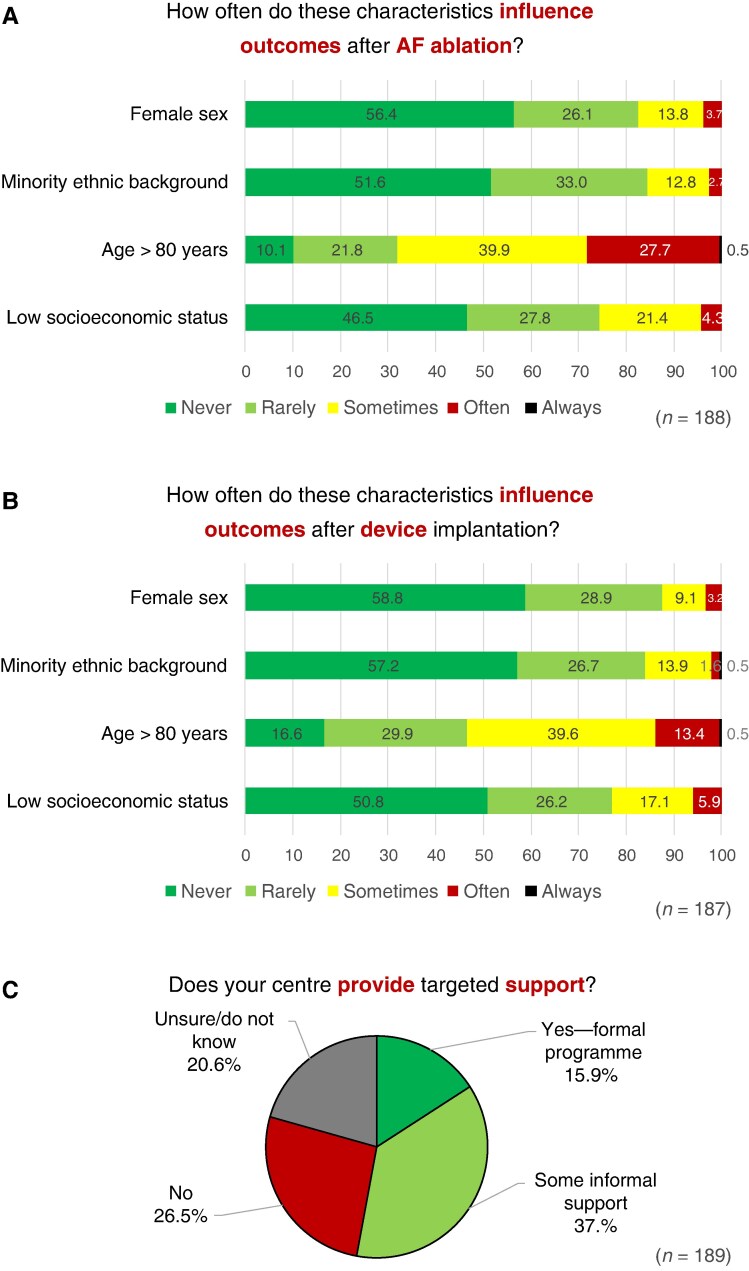
Follow-up and outcomes. (*A*) Impact of patient characteristics after AF ablation. (*B*) Impact of patient characteristics after device implantation. (*C*) Targeted support to patients who may face barriers to accessing healthcare after ablation or device procedures. AF, atrial fibrillation.

Age >80 years was most frequently reported to influence outcomes following AF ablation, affecting outcomes sometimes, often, or always in 68.1% of responses (*Figure 6A*). Lower proportions reported differences related to low socioeconomic status (25.7%), female sex (17.6%), and minority ethnic background (15.4%) (*Figure 6A*). A similar pattern was observed following device implantation, where age influenced outcomes in 53.5% of responses (*Figure 6B*).

Post-procedural support was variable, with 15.9% of respondents reporting formal targeted programmes and 37.0% informal support, while 26.5% reported no structured support and 20.6% were unsure (*Figure 6C*).

### Institutional policy and future priorities

Only 25.1% of respondents reported that their institution had a formal diversity, equity, and inclusion policy, while 41.0% reported none and 33.9% were unsure. Active institutional measures to address disparities were reported by 15.2% of respondents.

**Figure 7:**
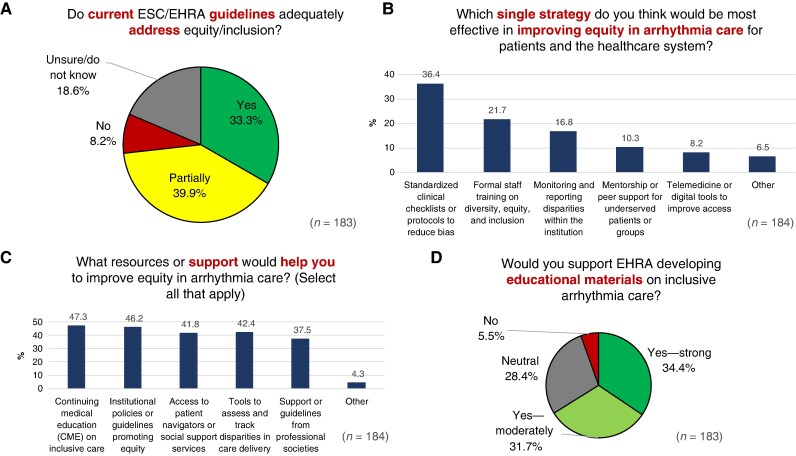
Institutional policy and future priorities. (*A*) Perception of adequacy of current arrhythmia guidelines. (*B*) Strategies to improve equity in arrhythmia care. (*C*) Resources to support clinicians in improving equity in arrhythmia care. (*D*) Support for the development of educational EHRA materials. EHRA, European Heart Rhythm Association; ESC, European Society of Cardiology.

Only one-third of respondents reported that current European Society of Cardiology (ESC)/EHRA guidelines adequately address equity and inclusion in arrhythmia care, whilst the remainder reported partial or inadequate coverage or were unsure (*Figure 7A*).

Standardized clinical protocols or checklists were most frequently identified as the potentially most effective strategy to improve equity (36.4%), followed by staff training (21.7%), and monitoring of disparities (16.8%) (*Figure 7B*). Commonly identified resources that might help respondents improve equity in arrhythmia care included continuing medical education (47.3%), institutional policies (46.2%), tools to assess disparities (42.4%), and patient navigation or support services (41.8%) (*Figure 7C*).

There was strong support for further educational initiatives, with approximately two-thirds of respondents supporting EHRA-led educational materials (*Figure 7D*).

### Perceptions according to respondent gender

**Figure 8:**
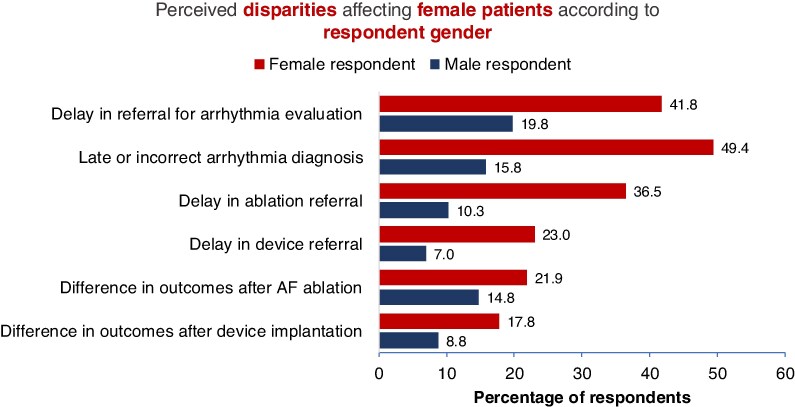
Differences in perceived disparities affecting female patients according to respondent gender. Values represent the proportion of respondents reporting disparities sometimes, often, or always. Analyses were stratified by self-reported respondent gender (male vs. female); the single respondent identifying as non-binary was excluded from this analysis because meaningful sub-group comparison was not possible. AF, atrial fibrillation.

When stratified by respondent gender, female respondents were consistently more likely than male respondents to report disparities affecting female patients across multiple domains of arrhythmia care (*Figure 8*). The largest differences were observed in diagnostic and referral domains. Defining a positive response as reporting disparities sometimes, often, or always, female respondents reported disparities in delayed referral for arrhythmia evaluation in female patients more frequently than male respondents (41.8 vs. 19.8%; absolute difference +22.0%), and were substantially more likely to report late or incorrect diagnosis (49.4 vs. 15.8%; +33.6%). Similar patterns were observed for procedural referral. Female respondents reported delayed referral for catheter ablation in female patients in 36.5% of responses compared with 10.3% among male respondents (+26.2%), and delayed referral for device implantation in 23.0 vs. 7.0% (+16.0%).

In contrast, differences in perceived disparities in procedural outcomes were smaller. Female respondents reported differences in outcomes following AF ablation in female patients in 21.9% of responses compared with 14.8% among male respondents (+7.1%), and differences following device implantation in 17.8 vs. 8.8% (+9.0%).

## Discussion

Over recent years, cardiac arrhythmia care has advanced substantially, driven by expanding evidence on disease mechanisms and novel therapeutic approaches. Innovative technologies have been increasingly adopted with the aim of improving the efficacy and safety of arrhythmia therapy. However, in everyday clinical practice, these optimized treatment options may not be equally accessible to all patient sub-groups. Disparities and inequities in cardiovascular care have been recognized, including in the management of cardiac arrhythmias.^1–4^

In this international survey supported by EHRA, we identified perceived disparities in the access to and delivery of arrhythmia care across several patient-related domains, most notably age, cognitive impairment, socioeconomic status, and sex. Respondents perceived that vulnerable sub-groups may encounter barriers at multiple stages in arrhythmia care, including screening, diagnosis, referral, treatment stratification, and outcome. These findings suggest that, despite substantial progress in arrhythmia management and the broad availability of modern technologies, equal access to contemporary and guideline-directed therapy is not yet fully achieved in routine clinical practice.

Age has been identified as affecting arrhythmia care on multiple levels of the arrhythmia care pathway. Physicians perceived differences in arrhythmia screening and strategies in AF-treatment, with rate control being preferred over rhythm control, particularly with catheter ablation, in older patients. This is in line with previous epidemiological studies.^10^ In this survey, age was perceived as one of the main influencing factors with respect to outcomes after catheter ablation. However, contemporary evidence and real-world data show that AF ablation in selected elderly patients is safe and efficient with modern ablation technologies.^18–20^ This stands in contrast with the perceived age-related disparities identified in this survey, which highlights the need for promoting individualized therapy stratification taking all patient-related characteristics and preferences into account, rather than relying exclusively on age.

Cognitive impairment has been described as affecting treatment decisions in arrhythmia care, specifically in AF.^21^ In the SAGE-AF study, cognitive impairment was associated with the preferential use of a rate control strategy, even though rate control rather than rhythm control was associated with further cognitive decline.^22^ These findings are in line with our survey and underscore the complexity of treatment stratification in this population.

The survey also highlights the role of social and economic determinants of arrhythmia therapy. Low socioeconomic status, minority ethnic background, non-native language, and immigration or residency status were all perceived to affect care, particularly in screening, therapy adherence, and referral for invasive therapies.^2^ These findings are consistent with previous reports showing that limited health literacy, reduced access to specialty care, communication barriers, and system-level factors can all affect access to appropriate therapy.^14,23^

The influence of sex on arrhythmia care was described as relevant in about a quarter of respondents. A considerable proportion of respondents perceived that women experience disadvantages in arrhythmia care, especially regarding screening, diagnosis, and referral for ablation. This is consistent with multiple real-world studies reporting that women are referred for ablation therapies less often and at a later stage of the disease.^14,24–26^ They more often receive pharmacological treatment, even though women are particularly prone to side-effects of anti-arrhythmic drugs.^27^ Emerging evidence also suggests that women may be less likely to receive oral anticoagulation despite appropriate indications, particularly at older ages, highlighting that sex-related differences in AF management extend beyond rhythm-control strategies.^28^ After AF ablation, women more often experience arrhythmia recurrence or procedural complications.^9,25,29–31^ The reason for limited therapeutic success of AF ablation in women is incompletely understood. However, later referral and more advanced atrial remodelling at the time of ablation therapy, as well as differences in symptom recognition, may contribute to this observation.^30^ It is noteworthy that the role of sex in arrhythmia care is evolving in certain areas, including stroke risk assessment. Contemporary ESC guidelines now recommend the CHA_2_DS_2_-VA score, reflecting evidence that female sex may function more as a risk modifier than an independent stroke risk factor.^32,33^ Importantly, this concept is distinct from the sex-related disparities in diagnosis, referral, and treatment explored in the present survey.

Importantly, the present survey revealed that female and male physicians may assess sex-specific disparities in arrhythmia care differently. Female physicians more often recognized deficits in arrhythmia screening, correct diagnosis, and timely referral in female patients. This is in line with previous reports describing common under- or misdiagnosis of arrhythmia symptoms in women.^34,35^ Furthermore, female respondents more often perceived later referral for AF ablation in women. The sex-specific sub-analysis of this survey highlights differences in clinician perceptions regarding arrhythmia care in female patients between female and male physicians. In acute coronary syndrome, female patients have been shown to have better outcomes when treated by female rather than male physicians, highlighting a potential sex-related bias in symptom assessment and treatment with prognostic relevance for the affected patients.^36^ The results of our survey corroborate the hypothesis that physician-related factors, including sex-specific perception of disease presentation and care pathways, may influence the recognition of inequities in arrhythmia management. Whether these sex-related perceptual differences among physicians in the present survey translate into measurable differences in patient outcomes in cardiac arrhythmia warrants further investigation. Nevertheless, these findings highlight an area where perceptions of inequity differ between clinicians and may warrant further investigation using objective patient-level data.

This survey further reveals a lack of formal training as well as a lack of institutional policies regarding topics of diversity, equity, and inclusion, as reported by most respondents. Only one-third of respondents stated that current guidelines adequately reflect these topics and the clinical reality related to it. The reason for this deficit in structured programmes and recommendations may lie in the lack of scientific evidence with respect to vulnerable populations and the absence of validated protocols for use in routine clinical practice.

In summary, these findings imply that while awareness of inequity and vulnerabilities of certain patient sub-groups exists, it has not yet been translated into established institutional processes. Standardized protocols, staff training, and routine monitoring of disparities were the most frequently endorsed solutions. This indicates that clinicians advocate society- or institution-based interventions rather than individual effort to address inequity in arrhythmia care. Thus, the results of the present survey suggest a need for educational and institutional initiatives aimed at addressing perceived inequities in arrhythmia care and improving awareness of potential disparities. Furthermore, this goal should be supported by future research specifically including or focusing on vulnerable patient subpopulations for the evaluation of modern arrhythmia therapies.

### Limitations

This study is subject to limitations inherent to survey design. Despite receiving responses from a broad geographic distribution, the survey is subject to selection bias; respondents with a particular interest in diversity, equity, and/or inclusion may have been more likely to participate, potentially limiting generalizability. Importantly, this survey assessed clinician perceptions rather than objectively measured patient-level disparities; therefore, the findings should not be interpreted as direct evidence of inequities in care delivery or clinical outcomes. Most answers were obtained from a subgroup of European countries, and some healthcare systems may therefore be under-represented. Furthermore, most respondents were characterized by considerable clinical experience and working at high-volume, metropolitan centres. Accessibility of modern arrhythmia therapy and, thus, equality in arrhythmia treatment may be different in less specialized settings, as well as in more rural regions, which may be under-represented in this respondent base. Nevertheless, the reported patterns are consistent over the various aspects of arrhythmia care, reflecting a reliable and clinically relevant observation.

## Conclusions

This international survey indicates that many physicians perceive substantial inequities in arrhythmia care across different European healthcare systems. Older patients, women, cognitively impaired individuals, and socially disadvantaged persons were commonly perceived as vulnerable patient sub-groups. Female physicians were more likely than male physicians to report perceived disparities affecting female patients across the arrhythmia care pathway. These findings support further investigation of potential disparities in arrhythmia care and highlight opportunities for educational and guideline initiatives aimed at promoting equitable care.

## Supplementary Material

euag144_Supplementary_Data
