# Supplementary material for: Diversity, equity, and inclusion in arrhythmia care: a European Heart Rhythm Association survey
Source: Europace. 2026 Jun 11;28(6):euag144. doi: 10.1093/europace/euag144 (PMC13318158; doi:10.1093/europace/euag144)
Supplement: euag144_Supplementary_Data [file euag144_supplementary_data.docx]

**Supplementary Material – Appendix 1: Full survey**

| **Diversity, equity and inclusion in arrhythmia care: a European Heart Rhythm Association survey** |
| --- |

# Introduction

Equity and inclusion are important considerations in delivering high-quality, patient-centred healthcare. In the field of cardiac electrophysiology (EP), there is increasing interest in whether and how potential differences between groups may influence access to arrhythmia care, treatment decisions and/or clinical outcomes.

This EHRA Scientific Initiatives Committee survey seeks to explore clinician experiences and perceptions regarding potential differences in arrhythmia care across a range of patient characteristics, including sex, gender identity, ethnicity, language, age and socioeconomic status. It also aims to assess how institutions are responding to these issues and what role professional societies might play in supporting inclusive and equitable care.

We invite all professionals involved in arrhythmia care (including electrophysiologists, general cardiologists, trainees and allied health professionals) to share their insights. The survey addresses the entire patient journey, from initial assessment and diagnosis through to pharmacological and invasive treatment, and long-term follow-up.

Your responses will help EHRA gain a more complete understanding of current practice, identify areas where guidance or support may be helpful and inform future educational initiatives. The survey takes approximately 10–12 minutes to complete. All responses are anonymous and all information provided in this study will be treated confidentially.

Thank you for contributing to this initiative.

Disclaimer

Your participation is anonymous.

We will not disclose your identity to any third party.

We comply with the European General Data Protection Regulation (GDPR) 2016/679. Any personal data processed in connection with this survey will be treated confidentially and only used by the ESC for the purposes of market research and not for promotion. Survey results will be kept for a maximum of 48 months for analysis and quality control purposes. We take all reasonable care to prevent any unauthorised access to your personal data. We respect your privacy and your right to access, modify, or remove your personal data. At any time, you can ask to know what personal data is being held. If you have any questions about data protection or require further information, please contact our data protection officer (DPO) at dpo@escardio.org.

You have the right to end your participation in this survey at any time.

**Please confirm that you have read the above and agree to participate in this survey.**

☐ Yes

☐ No

# Section 1: Respondent characteristics

1. Country of practice

☐ Dropdown list

1. What is your current role in arrhythmia care?
   ☐ Physician – consultant electrophysiologist and/or device specialist
   ☐ Physician – other/referring consultant cardiologist or physician involved in arrhythmia care
   ☐ Physician – fellow / trainee
   ☐ Nurse
   ☐ Physiologist / technician
   ☐ Other: free text
2. Years in arrhythmia practice:
   ☐ <5 years
   ☐ 5–10 years
   ☐ 11–20 years
   ☐ >20 years
3. Primary institution type:
   ☐ Academic/university hospital
   ☐ Public/teaching hospital
   ☐ District general hospital
   ☐ Private hospital or clinic

☐ Ambulatory/community setting
☐ Other: free text

1. **Do you primarily work in a metropolitan/urban area or a rural area?**
   ☐ Metropolitan/urban
   ☐ Rural
   ☐ Mixed/both
2. Your gender:
   ☐ Female
   ☐ Male
   ☐ Non-binary
   ☐ Prefer not to say

# Section 2: Perceptions of equity in arrhythmia care

1. How often do the following patient factors affect the overall provision of arrhythmia care (including diagnosis, treatment and follow-up) in your region?

| **Factor** | **Never** | **Rarely** | **Sometimes** | **Often** | **Always** |
| --- | --- | --- | --- | --- | --- |
| Female sex (biological) |  |  |  |  |  |
| Gender identity |  |  |  |  |  |
| Minority ethnic background |  |  |  |  |  |
| Non-native speaker |  |  |  |  |  |
| Cultural and/or religious beliefs |  |  |  |  |  |
| Age >80 years |  |  |  |  |  |
| Cognitive impairment |  |  |  |  |  |
| Mental health disease |  |  |  |  |  |
| Low socioeconomic status |  |  |  |  |  |
| Educational background |  |  |  |  |  |
| Immigration or residency status |  |  |  |  |  |
| Neurodiversity (e.g. autism) |  |  |  |  |  |
| Digital literacy |  |  |  |  |  |
| Type of health insurance |  |  |  |  |  |
| Rural or geographically isolated area |  |  |  |  |  |

1. Have you received any training in health equity or inclusive care delivery?
   ☐ Yes – formal course or module
   ☐ Yes – informal
   ☐ No
   ☐ Unsure / do not know

# Section 3: Diagnosis and screening

1. In your experience, do you observe differences in how often patients are referred for arrhythmia evaluation among the following patient cohorts?

| **Cohort** | **Never** | **Rarely** | **Sometimes** | **Often** | **Always** |
| --- | --- | --- | --- | --- | --- |
| Female sex |  |  |  |  |  |
| Minority ethnic background |  |  |  |  |  |
| Age >80 years |  |  |  |  |  |
| Low socioeconomic status |  |  |  |  |  |

1. In your experience, do you observe differences in access to ambulatory ECG monitoring (e.g. Holter, implantable loop recorder) among the following patient cohorts?

| **Cohort** | **Never** | **Rarely** | **Sometimes** | **Often** | **Always** |
| --- | --- | --- | --- | --- | --- |
| Female sex |  |  |  |  |  |
| Minority ethnic background |  |  |  |  |  |
| Age >80 years |  |  |  |  |  |
| Low socioeconomic status |  |  |  |  |  |

1. In your experience, do you observe differences in rates of late or incorrect diagnosis of arrhythmia among the following patient cohorts?

| **Cohort** | **Never** | **Rarely** | **Sometimes** | **Often** | **Always** |
| --- | --- | --- | --- | --- | --- |
| Female sex |  |  |  |  |  |
| Minority ethnic background |  |  |  |  |  |
| Age >80 years |  |  |  |  |  |
| Low socioeconomic status |  |  |  |  |  |

1. Does your centre routinely offer interpreter services for non-native speakers?
   ☐ Always
   ☐ Often
   ☐ Sometimes
   ☐ Rarely
   ☐ Never

☐ Unsure / do not know

# Section 4: Pharmacological management

1. Do you tailor anti-arrhythmic drug choice based on patient sex?
   ☐ Yes – routinely
   ☐ Yes – in selected cases (e.g. QTc)
   ☐ No
   ☐ Unsure
2. In your experience, does a patient’s background affect your choice between prescribing a rhythm-control drug or a rate-control drug as first-line therapy for atrial fibrillation among the following cohorts?

| **Cohort** | **Never** | **Rarely** | **Sometimes** | **Often** | **Always** |
| --- | --- | --- | --- | --- | --- |
| Female sex |  |  |  |  |  |
| Minority ethnic background |  |  |  |  |  |
| Age >80 years |  |  |  |  |  |
| Low socioeconomic status |  |  |  |  |  |

1. In your experience, how much does a patient’s background influence your decision to prescribe a **rhythm-control** versus a **rate-control** drug as first-line therapy for atrial fibrillation in the following groups?

| **Cohort** | **Never** | **Rarely** | **Sometimes** | **Often** | **Always** |
| --- | --- | --- | --- | --- | --- |
| Female sex |  |  |  |  |  |
| Minority ethnic background |  |  |  |  |  |
| Age >80 years |  |  |  |  |  |
| Low socioeconomic status |  |  |  |  |  |

1. In your experience, how often do the following patient characteristics affect adherence to atrial fibrillation medications?

| **Cohort** | **Never** | **Rarely** | **Sometimes** | **Often** | **Always** |
| --- | --- | --- | --- | --- | --- |
| Female sex |  |  |  |  |  |
| Minority ethnic background |  |  |  |  |  |
| Age >80 years |  |  |  |  |  |
| Low socioeconomic status |  |  |  |  |  |

# Section 5: Procedural referral and selection

1. In your experience, among patients eligible for catheter ablation, how often are the following groups **less likely** to be referred, or experience delayed referral, compared with other patients?

| **Cohort** | **Never** | **Rarely** | **Sometimes** | **Often** | **Always** |
| --- | --- | --- | --- | --- | --- |
| Female sex |  |  |  |  |  |
| Minority ethnic background |  |  |  |  |  |
| Age >80 years |  |  |  |  |  |
| Low socioeconomic status |  |  |  |  |  |

1. In your experience, among patients eligible for device implantation (e.g. pacemaker, cardiac resynchronisation therapy, implantable cardioverter defibrillator), how often are the following groups less likely to be referred, or experience delayed referral, compared with other patients?

| **Cohort** | **Never** | **Rarely** | **Sometimes** | **Often** | **Always** |
| --- | --- | --- | --- | --- | --- |
| Female sex |  |  |  |  |  |
| Minority ethnic background |  |  |  |  |  |
| Age >80 years |  |  |  |  |  |
| Low socioeconomic status |  |  |  |  |  |

1. In your experience with your patients, do their cultural or religious beliefs commonly influence decisions regarding catheter ablation or device implantation?
   ☐ Yes – frequently
   ☐ Occasionally
   ☐ Rarely
   ☐ Never
2. Before performing catheter ablation or a device implantation procedure, do you adjust your procedural counselling or technique based on patient sex?
   ☐ Never
   ☐ Rarely
   ☐ Sometimes
   ☐ Often

☐ Always

1. In your experience, have you observed higher complication rates after catheter ablation or device implantation in any of the following patient groups? (*Select all that apply)*

☐ Female patients (biological sex)
☐ Patients from an ethnic minority background
☐ Patients aged >80 years
☐ Patients with low socioeconomic status

# Section 6: Follow-up and monitoring

1. In your experience, how often do the following patient characteristics influence clinical outcomes (e.g. procedural safety, acute procedural success and/or long-term arrhythmia-free survival) after catheter ablation of atrial fibrillation?

| **Cohort** | **Never** | **Rarely** | **Sometimes** | **Often** | **Always** |
| --- | --- | --- | --- | --- | --- |
| Female sex |  |  |  |  |  |
| Minority ethnic background |  |  |  |  |  |
| Age >80 years |  |  |  |  |  |
| Low socioeconomic status |  |  |  |  |  |

1. In your experience, how often do the following patient characteristics influence clinical outcomes (e.g. procedural safety, acute procedural success) after device implantation?

| **Cohort** | **Never** | **Rarely** | **Sometimes** | **Often** | **Always** |
| --- | --- | --- | --- | --- | --- |
| Female sex |  |  |  |  |  |
| Minority ethnic background |  |  |  |  |  |
| Age >80 years |  |  |  |  |  |
| Low socioeconomic status |  |  |  |  |  |

1. Does your centre provide targeted support to patients who may face barriers to accessing healthcare after ablation or device procedures?
   ☐ Yes – formal programme (if selected, please specify targeted patient group(s) below in free-text box)
   ☐ Some informal support
   ☐ No
   ☐ Unsure / do not know

☐ Free-text box

# Section 7: Institutional policy and outlook

1. Does your hospital or arrhythmia department have a formal diversity, equity and inclusion policy?
   ☐ Yes
   ☐ No
   ☐ Not sure / do not know
2. To your knowledge, has your centre taken any specific steps to address disparities in access to arrhythmia care?
   ☐ Yes
   ☐ No
   ☐ Not sure / do not know
3. Which SINGLE strategy do you think would be most effective in improving equity in arrhythmia care **for patients** and the healthcare system? *(Select one)*

☐ Formal staff training on diversity, equity and inclusion

☐ Standardised clinical checklists or protocols to reduce bias

☐ Monitoring and reporting disparities within the institution

☐ Telemedicine or digital tools to improve access

☐ Mentorship or peer support for underserved patients or groups

☐ Other: free text

1. What resources or support would **help you** to improve equity in arrhythmia care? *(Select all that apply)*
   ☐ Continuing medical education (CME) on inclusive care
   ☐ Institutional policies or guidelines promoting equity
   ☐ Access to patient navigators or social support services
   ☐ Tools to assess and track disparities in care delivery
   ☐ Support or guidelines from professional societies
   ☐ Other: free text
2. Do you feel that current European Society of Cardiology (ESC)/European Heart Rhythm Association (EHRA) guidelines adequately address equity and inclusion in arrhythmia care?
   ☐ Yes
   ☐ Partially
   ☐ No
   ☐ Unsure / do not know
3. Would you support the EHRA developing educational materials on inclusive arrhythmia care?
   ☐ Yes – strongly
   ☐ Yes – moderately
   ☐ Neutral
   ☐ No
